# Mapping culturally sensitive assessment and care for Moroccan migrants with dementia in Spain

**DOI:** 10.1002/alz.086802

**Published:** 2025-01-09

**Authors:** Mohamed Taiebine

**Affiliations:** ^1^ Euro‐Mediterranean University of Fez (UEMF), Fez, Fez Morocco

## Abstract

**Background:**

Given the context of globalization, epidemiological and health disparities create socio‐cultural barriers to specialized and appropriate care for people living with dementia in non‐Western countries, particularly in Spain, where Moroccan migrants maintain their position as the largest registered foreign community. We aimed to map cultural, clinical, and linguistic challenges and facilitators of dementia assessment and care for this population.

**Methods:**

We investigated articles published in English, Spanish, and Arabic in different medical, allied health, social, and human sciences databases that addressed assessment and care for Moroccan migrants with dementia (MMWD) and their caregivers in Spain. Demographic, epidemiological, clinical, linguistic, socio‐cultural, and socio‐economic features as well as facilitators and barriers to assessment and care were summarized.

**Results:**

The main findings emphasized the importance of embracing the socio‐cultural values of the host country, as well as maintaining bi‐cultural and bilingual status. The principal challenges included lack of translating and interpreting services, paucity of neurocognitive tools in Moroccan Arabic, and dearth of bi‐cultural and bilingual practitioners, while the facilitators were mainly the training of healthcare staff about dementia and capitalizing on cross‐cultural translation and adaptation of best evidence‐based interventions and assessment tools in dementia in Moroccan Arabic and Spanish.

**Conclusions:**

Enabling cultural appreciation, inclusion and cross‐cultural competency should encourage stakeholders, practitioners, caregivers, and clients to respond culturally and linguistically to the unique features of elderly Moroccan Arabic migrants or those who are well‐established as Moroccan‐Spanish citizens. Implementing person‐centered care and culturally salutogenic approaches might erase frontiers in language, care, culture, and place of origin.

